# BrainNet Europe’s Code of Conduct for brain banking

**DOI:** 10.1007/s00702-014-1353-5

**Published:** 2015-01-13

**Authors:** Natasja M. Klioueva, Marleen C. Rademaker, David T. Dexter, Safa Al-Sarraj, Danielle Seilhean, Nathalie Streichenberger, Peer Schmitz, Jeanne E. Bell, James W. Ironside, Thomas Arzberger, Inge Huitinga

**Affiliations:** 1 Netherlands Brain Bank, Netherlands Institute for Neuroscience, Meibergdreef 47, 1105 BA Amsterdam, The Netherlands; 2 Division of Brain Sciences, Department of Medicine, Centre for Neuroinflammation and Neurodegeneration, Imperial College London, London, UK; 3 Department Clinical Neuropathology, London Institute of Psychiatry, London, UK; 4 Laboratoire de Neuropathologie, Raymond Escourolle, Université Pierre et Marie Curie and INSERM, Paris, France; 5 Service de Neuropathologie, Groupement Hospitalier Est, Hospices Civils de Lyon, Université Lyon 1, CNRS UMR5292, INSERM U1028, Lyon, France; 6 Centre for Neuropathology and Prion Research, Ludwig-Maximilians-University Munich, Munich, Germany; 7 Department of Pathology, University of Edinburgh, Western General Hospital, Edinburgh, UK; 8 Department of Psychiatry and Psychotherapy, Ludwig-Maximilians-University Munich, Munich, Germany

**Keywords:** Bioethics, Brain autopsy, Brain bank, Informed consent, Biobanking, Neuropathology

## Abstract

Research utilizing human tissue and its removal at post-mortem has given rise to many controversies in the media and posed many dilemmas in the fields of law and ethics. The law often lacks clear instructions and unambiguous guidelines. The absence of a harmonized international legislation with regard to post-mortem medical procedures and donation of tissue and organs contributes to the complexity of the issue. Therefore, within the BrainNet Europe (BNE) consortium, a consortium of 19 European brain banks, we drafted an ethical Code of Conduct for brain banking that covers basic legal rules and bioethical principles involved in brain banking. Sources include laws, regulations and guidelines (Declarations, Conventions, Recommendations, Guidelines and Directives) issued by international key organizations, such as the Council of Europe, European Commission, World Medical Association and World Health Organization. The Code of Conduct addresses fundamental topics as the rights of the persons donating their tissue, the obligations of the brain bank with regard to respect and observance of such rights, informed consent, confidentiality, protection of personal data, collections of human biological material and their management, and transparency and accountability within the organization of a brain bank. The Code of Conduct for brain banking is being adopted by the BNE network prior to being enshrined in official legislation for brain banking in Europe and beyond.

**Electronic supplementary material:**

The online version of this article (doi:10.1007/s00702-014-1353-5) contains supplementary material, which is available to authorized users.

## Introduction

Neurological and psychiatric diseases put a high toll on public and personal health and cause a heavy economic burden (Wittchen et al. 2011; Smith 2011). The pathological processes involved in neurological diseases such as Alzheimer’s and Parkinson’s disease are now becoming better understood. However, effective treatments and prevention measures are yet to be identified (Montine et al. 2012). In psychiatric diseases, the nature of molecular and cellular changes of the brain is still obscure (Deep-Soboslay et al. 2011). Notwithstanding recent impressive progress in MRI, genetic and biomarker studies as well as experimental animal research, validation by histological, cellular and molecular research of the human brain is still required (Lin et al. 2011). State of the art imaging techniques, genomics and proteomics make human brain tissue research more compelling than ever. However, CNS tissue samples can generally only be obtained after death and to foster research in human brain diseases, brain banks have been established worldwide (Kretzschmar 2009, Samarasekera et al. 2013).

Post-mortem removal and storage of human organs, research with human brain tissue and genetic research have often been the centre of media attention (Burton et al. 2003) and posed a great deal of questions in the fields of law and ethics (Andrews 2006; Case Law 1988; Greenfield 2006). Due to the relative novelty of the Brain Bank as an entity, the law is often lacking in clear instructions or specific guidelines. Frequently, brain banks do not fit into well-defined and harmonized legal regimes while existing local legal regimes applicable to donation and use of human organs and tissues for research purposes differ significantly between countries. Therefore, many uncertainties arise pertaining to initiation and governance of the brain banks, informed consent, donor confidentiality or tissue commercialization, which hampers the establishment of the valuable resources (Bell et al. 2008).

## The development of the Code of Conduct for brain banking and its foundations

The partners of the Brain Net Europe (BNE) consortium, a consortium of 19 European brain banks, belong to a variety of academic and/or health institutions, established in eleven different European countries. All of the BNE Brain Banks are either affiliated with or are part of (neuro)pathology departments or neuroscience institutes. Considerable variations in practice, policies and legal requirements are inevitable. The lack of harmonization in European legislation with regard to post-mortem medical procedures and donations of tissue to a biobank for research purposes contributes to the complexity of the field. Although a set of uniform exhaustive operating requirements was deemed unfeasible to formulate and perhaps in many cases undesirable to impose, we reasoned that a certain set of minimum ethical standards had to be maintained at all times, regardless of the differences among the brain banks. To formulate these standards, we systematically surveyed various authoritative sources in the disciplines of law and ethics. These include Declarations, Conventions, Recommendations, Guidelines and Directives issued by the international governmental and non-governmental organizations, such as the Council of Europe, European Commission, World Medical Association and World Health Organization (documents used in the preparation of the Code of Conduct see: http://www.brainnet-europe.org/index.php?option=com_content&view=article&id=104&Itemid=104%29, http://www.brainbank.nl).

Specifically, the Recommendation on Research on Biological Materials of Human origin adopted by the Committee of Ministers of the Council of Europe was important in the design of the Code of Conduct and functioned as a template for its first draft. To establish a common ground as well as fundamental differences in the practices and the legal frameworks under which brain banks operate, and to involve more BNE members in the development of the first drafts of the code of conduct, a meeting in Amsterdam was organized in November 2006. During this meeting, attendees from different BNE brain banks have deliberated on principal points to be included in the code of conduct, thereby setting a certain course. The first draft of the Code of Conduct was presented during a BNE meeting in Stockholm, in June 2007. The discussions during this meeting had mainly revolved around the requirement for informed consent. Although it had been established that informed consent of the donor or the next of kin is not always prescribed by law for conducting an autopsy and retaining whole organs or tissue samples for examination or research purposes, the BNE members agreed that a brain bank should strive to implement the information and consent procedure in their daily practice (Lunetta et al. 2007; Sakr et al. 1989). By this “declaration of intention” in the code of conduct, an aim for a future standard had been set. Following this meeting, the final draft of the Code of Conduct has been circulated among all BNE members with a possibility to comment on its contents and suggest textual amendments. During the next meeting in Barcelona in June 2008, the final version of the Code of Conduct was ratified by all BNE brain banks. Subsequently, at an International Conference on Brain Banking, organized by the BNE network, in Munich in December 2008 (http://www.brainnet-europe.org/index.php?option=com_content&view=article&id=125&Itemid=141), a workshop on legal and ethical issues in Brain Banking was held where the applicability of the Code of Conduct was discussed. This workshop was attended by the directors of Brain Banks, scientists as well as representatives of patient organizations from many different countries. Practical examples of Code of Conduct application in daily practice were discussed.

## The contents of the Code of Conduct and its purpose

The Code of Conduct consists of four Chapters (for the text of BNE's Code of Conduct for brain banking see supplementary material). The first Chapter sets out the objectives, general principles and the scope of the Code of Conduct, as well as defining key terminology (indicated in the text by Capital Letters). The second Chapter deals with different aspects of Material procurement (e.g. Informed Consent, Authorization, Incompetent persons, Autopsy). The third Chapter states the principles that should govern the processing of the Material (e.g. financial aspects, confidentiality, data protection measures). The fourth Chapter is concerned with distribution and the use of the Material for research. It also contains articles on research results and guidelines on how to deal with information on hereditary diseases.

The Code of Conduct for brain banking states the principles that should govern brain banking in general. Detailed regulations concerning the local governance of a Brain Bank are to be laid down in a different document such as a Brain Bank Regulations, which should be in concordance with the principles described in the code of conduct. Furthermore to support the daily practice of the Brain Bank documents such as a Material Transfer Agreements, Confidentiality Agreements, Tissue application forms and Personal Data Protection measures should be in place. Examples of these documents are provided on the BNE website (http://www.brainnet-europe.org/index.php?option=com_content&view=article&id=103&Itemid=103). The scope of the Code of Conduct could be widened to cover all post-mortem tissue donations for research purposes, whether of CNS origin or not (for instance peripheral lymph nodes or dorsal root ganglia), since the same principles apply.

## Conclusion

BNE’s Code of Conduct is being adopted by the BNE network prior to being enshrined in official legislation. BNE has no legal power to enforce it. However, this Code of Conduct is a first common European attempt to define ethical standards in Brain Banking in one document. In case of BNE, the Code of Conduct has stimulated the BNE brain banks to reflect on their daily practice and on the governance of their brain banks. We hope that brain banking will receive more attention in future legislation, locally and internationally, and that this Code of Conduct may lay in part a foundation for more official legislation for brain banking in Europe and beyond.

## Electronic supplementary material

Below is the link to the electronic supplementary material.

Supplementary material 1 (120 kb)
