# Supplementary material for: BrainNet Europe’s Code of Conduct for brain banking
Source: J Neural Transm (Vienna). 2015 Jan 13;122(7):937–40. doi: 10.1007/s00702-014-1353-5 (PMC4498226; doi:10.1007/s00702-014-1353-5)
Supplement: Supplementary file 1 — Supplementary material 1 (120 kb) [file 702_2014_1353_MOESM1_ESM.pdf]

BRAINNET EUROPE

Code of Conduct

BNE Consortium
May 30th, 2008

Table of contents

PREAMBLE		2
CONSIDERATIONS		3
SIGNATURE PAGE		4
CHAPTER 1	Articles, definitions and scope	6
Article 1	Definitions	6
Article 2	Objectives and general principles	8
Article 3	Scope	8
Article 4	Brain Bank Regulations	9
CHAPTER 2	Obtaining of Human Material and associated Data for research	9
Article 5	Informed Consent and Authorization	9
Article 6	Obtaining of Informed Consent and Authorization	9
Article 7	Persons unable to give Informed Consent	10
Article 8	Rights to change the scope or to withdraw Consent or Authorization	10
Article 9	Autopsy	11
Article 10	Professional standards for autopsy and diagnostics	11
Article 11	Facilities for autopsy	12
Article 12	Residual Biological Materials	12
CHAPTER 3	Principles applicable to Brain Banking	12
Article 13	Prohibition of financial gain	12
Article 14	Confidentiality, Principles of Data Protection and Ownership of Data	12
Article 15	Data Protection Measures	13
Article 16	Directly Identifying Material and Data	14
Article 17	Protection of the electronically processed data	14
Article 18	Other principles applicable to Brain Banking activities	14
Article 19	Trans border flow	15
CHAPTER 4	Distribution and Use of Biological Materials for research projects	15
Article 20	Distribution of the Material	15
Article 21	Requests for Material	16
Article 22	Research Results	16
Article 23	Information on Hereditary Diseases	17

PREAMBLE

The Brain Network Europe Consortium, hereinafter "BNE Consortium", is comprised of eighteen full brain bank partners, one associated brain bank partner and a company for project, controlling and communication management, as defined in the BNE Consortium Agreement singed in September 30th, 2006. The BNE Consortium has been established in the 6th Framework project, funded by the European Commission under contract number LSHM-CT-2004-503039.

The Brain Banks which belong to BNE Consortium obtain, handle, process, store and distribute biological material of human origin and related data. The material is obtained through post-mortem autopsies, therapeutic interventions or diagnostic procedures and retained for research purposes. Ultimately the material and data are utilized for research in the field of neuroscience.

The objectives of the BNE Consortium are:
• to promote Brain Banking as a research resource for the European neuroscience community through the provision of high quality human Central Nervous System (CNS) material;
• to determine the effect of pre- and post mortem parameters on preservation of molecular subsets of the material such as DNA, RNA, proteins and neurochemical substances;
• to determine the limits of usability of human post-mortem CNS material for advanced molecular techniques;
• to develop gold standards for obtaining, processing and distributing of human CNS material, quality control and ethics leading to best practice guidelines for brain banking;
• to provide training in Brain Banking and related methodology;
• to reach out to neuroscience centers worldwide and promote future expertise in CNS research.

CONSIDERATIONS

Recalling the Convention for the Protection of Human Rights and Fundamental Freedoms of the Council of Europe, Rome, 4.XI.1950;

Recalling the Convention on Human Rights and Biomedicine and the additional protocols to the Convention of the Council of Europe, Oviedo, 4.IV.1997;

Recalling the Universal Declaration of Human Rights, 10.XII.1948 and the Universal Declaration on the Human Genome and Human Rights, 11.XI.1997 and the International Declaration on Human Genetic Data of the United Nations, 16.X.2003;

Recalling the Universal Declaration of Bioethical Principles of the United Nations 19.X.2005;

Recalling the Declaration of Helsinki of the World Medical Association, 1.VI.1964;

Recalling the Directive of the European Commission EC 98/44/EC on the Legal Protection of the Biotechnological Inventions;

Recalling the Directive of the European Commission EC 95/46/EC on Protection of Individuals with Regard to Processing of Personal Data and Free Movement of such Data;

Considering the Recommendation on Research on Biological Materials of Human Origin adopted by the Committee of Ministers of the Council of Europe, 15.III/2006, in particular instructions on establishing codes of good practice to ensure compliance with the provisions of the Recommendation laid down in art. 4,;

Considering the objectives laid down in the Consortium Agreement

CHAPTER 1

Articles, definitions and scope

Article 1 Definitions

Brainnet Europe II-project	Sixth Framework project Brainnet Europe II, funded by the European Commission under contract number LSHM-CT-2004-503039.
Consortium Agreement	Agreement signed on September 30, 2006 between participants in the BrainNet Europe II-project, by which BNE Consortium has been established.
BNE Consortium Partners	Partners of the BNE Consortium, who conduct Brain Banking Activities.
BNE Consortium Associated Partners	Allied partners without voting rights, who may participate in meetings and in activities of BNE Consortium without liability to the European Commission or any contractual duties.
Brain Banking/ Brain Bank Activities	Activities such as obtaining, handling, processing, storing and distributing of Material and Data including all associated research activities.
Informed Consent	Informed, free, express and documented decision to donate Material and Data for scientific research or other purposes.
Authorization	Informed Consent given by a legal representative or the next of kin on behalf of a person unable to give Informed Consent.
Brain Donation Program	A program, where a person can record his or her intent for donation or provide Informed Consent or provide Authorization for post-mortem donation of the Material and Data to the Brain Bank.
Human Biological Material	Human Biological Material including:<br>I. Tissues: brain and meninges, pituitary, spinal cord, peripheral nervous tissue and other tissues adjacent to the nervous system such as muscles, lymph nodes and blood vessels;<br>II. Fluids: such as cerebrospinal fluid or blood;<br>III. Cells derived from all of the above-mentioned, including their progeny and unmodified derivatives.
Donor	The source of the Biological Material.
Material and Data	Any Human Biological Material and Donor related Data.
Directly Identifying Material or Data.	Any Material or any Data which, alone or in combination with associated information, allow for the identification of the person concerned, without disproportionate efforts.
Pseudo Anonymised/ Coded Material or Data.	Any Material or any Data which allow for the identification of the person concerned by authorized individuals only through the use of a key.

Fully Anonymised/ Unidentifying Material or Data	Any Biological Material or Data which alone or in combination with associated information do not, allow for the identification of the person concerned, without disproportionate efforts.
Information on Hereditary Diseases	Any information which is either predictive of genetic disease or can serve to identify the person as a carrier of a gene responsible for a disease or detect a genetic predisposition or susceptibility to a disease, whereas scientific proof for validity of that information is present.
Data Processing Equipment	All equipment that reads or writes data from or to digital media.
Data Communications Equipment	All equipment that allows the transport of data to, from or across any local or remote digital network.
Tissue Finder/ Tissue Finder Database/ Information	Means an online database built to facilitate Material exchange among the BNE Consortium Partners. The Tissue Finder Database contains descriptions of Material available at the individual Brain Banks of the BNE Partners. Data stored in Tissue Finder Database is either Pseudo Anonymised or Fully Anonymised and does not, with reasonable efforts, allow identification of the Donors by the persons searching the Database. The Tissue Finder Database is only accessible via encrypted internet. Partners of the BNE Consortium can search for and place requests for Material, which may or may not be granted.
BNE Request Form	Online form by the means of which researchers can submit a request for Material and data to the BNE Consortium.
Commercial exploitation	The sale, lease or commercial licensing of the Material. Commercial exploitation shall also include uses of the Material to produce or manufacture products for general sale.

Article 2 Objectives and general principles

1. National laws or local regulations should be observed in all Brain Banking activities.

Objectives of the Code of Conduct

2. The objective of this Code of Conduct is to state the fundamental ethical principles which should govern all Brain Banking Activities.

3. Partners of the BNE Consortium shall observe that autonomy and confidentiality of the Donors and the right to private life of their families are respected. With regard to the deceased persons, the Partners of the BNE Consortium shall guarantee respect for the human remains and observance for the wishes previously expressed by the deceased, as well as consideration for the wishes expressed by the surviving family and next of kin.

4. The Partners of BNE Consortium agree that every person has the right to accept or refuse to contribute to scientific research and that no one should be required to contribute to it

5. The Partners of BNE Consortium agree that all appropriate measures need to be taken to avoid discrimination against, or stigmatization of, a person, family or group.

6. Brain Banks shall ensure that the collected Material and Data is appropriately and regularly used for the purpose for which it was donated.

7. The Partners of the BNE Consortium shall strive to promote and facilitate ethically acceptable and scientifically sound research.

8. Brain Banking Activities should only be perfomed in an ethically accepatable manner. Periodic independent assessement of the Brain Banking Activities by a designated ethics committee is desirable.

Article 3 Scope

Scope

1. This Code of Conduct shall apply to the full range of Brain Banking Activities conducted with Material and Data. Where collection of Human Material is accompanied by the processing of associated Identifying Data, including personal data of living individuals and medical records of the deceased Donors, the Code of Conduct shall equally apply to Brain Banking Activities involving such data. The availability of good quality clinical information about the Donor is indispensable for the adequate, future use of the Material.

Equal applicability

2. The Code of Conduct shall equally apply to the maintaining of collections of Human Biological Material that was originally removed for a purpose other than scientific research, such as Material that is removed for diagnostic purposes or through therapeutic interventions and is subsequently retained for research purposes .

Scope limitation

3. This Code of Conduct does not apply to embryonic tissue and germ line cells.

Article 4 Brain Bank Regulations

RESERVED

CHAPTER 2

Obtaining of Human Material and associated Data for research

Article 5 Informed Consent and Authorization

1. Biological Material and Data should be obtained and used for research purposes in accordance with the national laws and regulations and provisions of this chapter.

Equal Informed Consent policy

2. All partners of the BNE Consortium explicitly underline the importance of Informed Consent or Authorization in obtaining of Human Material and Data for research purposes. By the Network Decision on June 8, 2007, it has been determined that Human Material and Data exchanged within the BNE Consortium or requested through BNE Request Form must be obtained on the basis of Informed Consent or Authorization. This decision comes into force twelve months after the above-mentioned date.

Implementation intention

3. Partners of the BNE Consortium, who by national laws or regulations either are not required to obtain Informed Consent or Authorization, or are able to waive these requirements, shall be encouraged to bring their internal standard operating procedures in conformity with this Code of Conduct.

Article 6 Obtaining of Informed Consent and Authorization

1. Prior to giving Informed Consent or Authorization the person concerned should be offered appropriate information on the nature and purpose of the post-mortem interventions and retention of Biological Material and Data, to be stored at the Brain Bank.

Information requirements Consent

2. A person providing Informed Consent or Authorization is free to place restrictions on the use of the Biological Material and Data. The restrictions should always be documented, prior to coding or anonymisation of the Material and Data.

Freedom to place restrictions

3. The person registering with the Brain Donation Program should beforehand be given appropriate information on the consequences of such registration and should be prompted to discuss this decision with his or her family or in the absence of family members with a confidant.

Information requirements Donor Program registration

4. Selection criteria for participation in a Brain Donation Program and post-mortem donation should be clearly stated by the Brain Bank.

Stating selection and exclusion criteria

5. Each Informed Consent, Authorization or registration with a Brain Donation Program should be appropriately documented at the Brain Bank administration.

Proper administration of Consent

6. RESERVED: (to be included here: instruction to follow the guidelines in Chapter II of the BBR)

Article 7 Persons unable to give Informed Consent

Registration of minors with the Donor Program

1. Where, according to law, a minor *permanently* does not have the capacity to provide Informed Consent, the registration of a minor as a Donor and subsequent post-mortem removal of the Biological Material should only be carried out with the Authorization of his or her parent or legal guardian. The opinion of the minor should be taken into consideration as an increasingly determining factor in proportion to his or her age and degree of maturity, such in full accordance with national legislation and regulations.

Registration of incompetent adults with the Donor Program

2. Where, according to law, an adult *permanently* does not have the capacity to give Informed Consent because of a mental disability, a disease or for similar reasons, the registration of the incompetent person as a Donor and subsequent post-mortem removal of the Biological Material should only be carried out with the Authorization of his or her legal guardian or representative. The individual concerned should as far as possible be involved in the decision-making process .

Subsidiary mean use of Material of the incompetent persons

3. Biological Material of persons mentioned in paragraph 2 and 3 of this article should only be collected and used for research purposes, when such research addresses important public interest and the aims of the research, cannot be achieved otherwise through the use of Material obtained on the basis of Informed Consent given by a competent person.

Obligation to regard previously expressed wishes

4. Previously expressed wishes by the person, who at the time when Authorization is required, is not in a state to express his or her wishes, should always be taken into consideration.

5. RESERVED: (to be included instruction to follow the guidelines in Chapter II of the BBR).

Article 8 Rights to change the scope or to withdraw Consent or Authorization

Right to withdraw Consent and non-discrimination

1. The person giving Informed Consent retains the right to withdraw or alter the scope of the Informed Consent. The withdrawal or alteration of Informed Consent may not lead to any form of discrimination against that person, in particular regarding his or her medical treatment or care.

Right to withdraw Authorization and non-discrimination

2. Where Authorization has been given on behalf of a person who is not able to provide Informed Consent, the representative or the next of kin retains the right to withdraw or alter the scope of Authorization.

Proper disposal in case of withdrawal

3. After the Informed Consent or Authorization has been withdrawn, any Identifying or Pseudo-Anonymised Material and Data obtained and stored for research purposes prior to withdrawal, should be disposed of in an appropriate manner.

Record of previously given Consent or Authorization

4. Notwithstanding the above-mentioned, a record of Informed Consent or Authorization given in the past should be retained, as documentation of the period of time during which the Material and Data were legitimately used for research .

Proper administration of withdrawals

5. In order to comply with a request for withdrawal, all withdrawals of registrations with a Brain Donation Program, Informed Consent and Authorizations should be appropriately documented, to ensure traceability to related Material and Data.

Article 9 Autopsy

1. Biological Material should only be removed from the body of a deceased person through an autopsy carried out in full accordance with national laws and regulations.	Autopsy
2. In cases where death may be due to unnatural causes, the Biological Material should only be removed and retained for research purposes, if this does not impede the course of the investigation into these causes.	No-interference with investigation into unnatural causes of death
3. In cases where the deceased person is registered or considered as an organ donor for transplantation purposes, the Biological Material should only be removed and retained for research purposes, if it does not impede the course of the organ and tissue harvesting for transplantation purposes , unless Informed Consent or Authorization explicitly orders to do otherwise.	No-interference with organ donation for transplantation

Article 10 Professional standards for autopsy and diagnostics

1. Macroscopic and microscopic examination of the brain, spinal cord and other relevant structures should be performed by a qualified neuropathologist or histopathologist trained in pathology of the central nervous system. Diagnostic standards for neuropathology, developed by the BNE Consortium, should be applied.	Pathologist
2. Trainees and students may participate in the procedure of autopsy under the supervision of a trained pathologist. The supervising pathologist always remains responsible for the diagnostic conclusions drawn from the autopsy and pathology examination.	Trainees and students
3. Conclusions drawn from post-mortem examination should be laid down in a pathology report, a copy of which should be provided to the medical doctors involved in recent treatment and care for the deceased. The wishes of the surviving family of the Donor to either be informed of the results of the post-mortem examination or to not to be informed of these results should be respected.	Pathology report and distribution of the pathology report
4. When certain diagnostic examinations should be performed by a person with training and expertise in specialized areas, a pathologist must recognize the limitations of his expertise and, if a case goes beyond those limitations, must seek the assistance of an appropriate specialist.	Scope of competence
5. The reconstruction of the body of the deceased Donor should be of a high standard, so that it can be viewed after autopsy without causing distress to the next of kin. The pathologist is responsible for the quality of the reconstruction and, if and when these tasks are delegated, ensuring that the persons performing reconstruction are adequately skilled.	High standards for reconstruction of the body

Article 11 *Facilities for autopsy*

Facilities for autopsy

1. An autopsy should only to be carried out on the dedicated premises of a recognized medical or forensic institution or a public mortuary, which is accredited and equipped to facilitate invasive post-mortem examinations.

Health and safety at autopsy

2. Facilities where autopsies are performed should meet current health and safety standards. The pathologist should bring to the attention of the management board of the facility any defect in the premises or equipment preventing the safe performance of a high quality autopsy.

Preservation of research Material

3. During and immediately after the autopsy, sufficient equipment and manpower should be available in order to collect and preserve the Biological Material for further diagnostic examination and research purposes.

Article 12 *Residual Biological Material*

Biological Material obtained otherwise

Biological Material removed for purposes other than research purposes, for example Biological Material removed for diagnostic purposes or therapeutic intervention, should only be made available for research with appropriate Informed Consent or Authorization.

CHAPTER 3

Principles applicable to Brain Banking

Article 13 *Prohibition of financial gain*

Non-commerciality and non-profit organizations

1. The partners of the BNE consortium are convinced that the human body or its parts should not, as such, give rise to financial gain. Noting that Biological Material is donated by the persons for altruistic reasons, the Brain Banking Activities should be carried out solely by non-profit organizations .

Legitimate costs

2. Reimbursements for the costs incurred by the Brain Bank involved in obtaining, processing, storing and distributing of the Biological Material and Data and related further inquiring activities, do not constitute financial gain .

Non-commerciality in the process of transfer

3. Partners of the BNE Consortium shall observe that in the process of transfer of Material from Donor to scientific researcher the Material, as such, will not be used for Commercial Exploitation.

Article 14 *Confidentiality, Principles of Data Protection and Ownership of Data*

Confidentiality and protection of private life

1. All data in relation to Donors, their families, representatives, legal guardians, confidants or the next of kin should be collected and processed by the Brain Banks in accordance with principles of confidentiality and respect for private life.

2. Directly Identifying Data of all persons concerned should only be collected, stored and processed in accordance with national personal data protection laws, provisions of this chapter and the standard operating procedures of the university or institution where the Brain Bank resides. Partners of the BNE Consortium should, where necessary, notify local supervisory data protection representatives or national data protection authorities of the processing of personal data.

Processing of Directly Identifying Data

3. Where it concerns Identifying Data collected for Brain Banking, the Partners of the BNE consortium shall observe that

i. such Data is only collected and processed for legitimate purposes consistent with the objectives of the Brain Bank and not further processed if incompatible with these purposes;

ii. such Data is adequate, relevant and not excessive in relation to the purposes it has been collected for;

iii. such Data is accurate, kept up to date and kept in the form which permits identification for no longer than necessary.

4. Where it concerns processing of personal Identifying Data of the living individuals, these persons retain the rights to access and modify any personal data collected about them.

5. RESERVED (instructions to follow the guidelines in the Chapter III of the BBR.)

Article 15 Data Protection Measures

1. The Partners of the BNE should implement and maintain appropriate technical and organizational measures and provide the necessary means to safeguard the integrity of all Identifying Data and other data of a confidential nature. All Identifying Data should be protected against unauthorized perception, copying, unlawful disclosure, unauthorized alteration, destruction or accidental loss. Quality assurance measures, to ensure security and confidentiality, should be in place.

Obligation to implement and maintain data protection measures

2. Individuals involved in the processing of Identifying Material and Data should be bound by legal obligations of confidentiality, such as contractual obligations of non-disclosure or professional obligations of medical confidentiality.

Non-disclosure

3. All Identifying Material and Identifying Donor Data either must be Fully Anonymised or Pseudo Anonimised appropriately, before these can be utilized for research purposes.

Anonymisation and Coding

4. Identifying Material and Data or the key to the decoding of Coded Material and Data should only be accessible to designated employees of the Brain Bank or other authorized individuals, for a number of well defined legitimate purposes, under the condition of full compliance with paragraph 2 of this article. A key to decoding of Coded Material and Data should never be made available to persons utilizing this Material and Data for research.

Authorized individuals

5. Identifying Material and Data, should never be transferred to a third party, excepting the circumstances when the third party is legally entitled to request and to receive such Material and Data. The transfer of any Identifying Data should be authorised by the legal entity, such as university or academic institution where the brain bank resides.

Transfers of Identifiable Data

6. RESERVED: (instruction to follow the guidelines in Chapter III of the BBR)

Article 16 Directly Identifying Material and Data

Use of Directly Identifiable Data and Material

Under exceptional circumstances, Directly Identifying Material and Donor Data can be utilized for research purposes, provided that the Donor or his or her representative has given fully informed and explicit consent thereto. Research utilizing Directly Identifying Material and Data should only be undertaken, subject to the approval of a designated Research Ethics Committee.

Article 17 Protection of the electronically processed data

Electronically processed Data

1. Where Identifying Data is processed electronically the following technical and organizational measures should be implemented:

i. Unauthorized access to the Data Processing Equipment with which Identifying Data are processed should be prohibited and measures put in place to ensure its enforcement.
ii. It should be ensured that media carrying Identifying Data cannot be read, copied, modified or removed by unauthorized persons.
iii. Safeguards to avoid unauthorized entry into memory of the Data Processing Equipment, resulting in unwanted perception, modification or deletion of Identifying Data should be put into place.
iv. Safeguards to avoid the usage of the Data Processing Equipment via Data Communications Equipment by unauthorized persons should be put in place.
v. It should be ensured that authorized usage of the Data Processing Equipment allows the authorized individual to access only his or her authorized data and no more.
vi. It should be ensured that one can check and retrospectively determine, to which destinations Identifying Data has been sent via Data Communications Equipment.
vii. It should be ensured that one can reproduce and retrospectively deter mine which Identifying Data files or entries were created and modified at what time and by whom.
viii. It should be ensured that Identifying Data processed by a person authorized to use the relevant system is processed in the required way to achieve the desired objectives and no more.
ix. It should be ensured that measures are put in place to prohibit unauthorized reading, copying or modification of Identifying Data during communication of such data via networks.
x. The internal organizational structure should be structured and organized in such a way that it is able to comply with all of the above-mentioned data protection requirements.

2. Paragraph 1, with exception of vii and viii, shall equally apply to electronic processing of other data of a confidential nature.

Article 18 Other principles applicable to Brain Banking activities

Transparency and accountability in Brain Bank management

1. The principles of transparency and accountability should govern the management of a Brain Bank, including access to, distribution and the use of its Biological Material and Data.

2. The governance of the Brain Bank should take place either by a management committee or under supervision of the management board, by persons with clear responsibilities and terms of reference.

3. The purposes of collection of Biological Material and Identifying Data should be specified and the objectives of the Brain Bank should be clearly defined.

Clear purposes and objectives

4. The person(s) and legal entity responsible for the collection of Biological Material and Data of a Brain Bank should be designated and delegated with well-defined tasks.

Responsible persons and organizations

5. Each sample of Biological Material in the collection of the Brain Bank should be appropriately documented, including information on any relevant Informed Consent or Authorization and any restrictions imposed.

Proper documentation of all Material

6. All stored Material including tissues, cells and fluids should be treated with the respect due to human Material, in a way that the dignity of the Donors is preserved.

Respect for human tissue

Article 19 Trans border flow

1. Material and Data should only be transferred to another country, in full accordance with national legislation and regulations, such as requirements to obtain a transfer permission from relevant national authorities, or requirement to obtain customs clearance for transfer of human tissue.

Trans border flow of Material and Data

2. Biological Material and Data shall only be transferred to another country if that state ensures an adequate level of protection.

3. Biological Material and Data transferred from another country should only be accepted if and when these have been obtained in conformity with the standards adequate to this Code of Conduct.

CHAPTER 4

Distribution and use of Material and Data for research projects

Article 20 Distribution of the Material

1. Distribution of the Material and Data should take place prudently, with due respect to the persons who have donated them for research purposes.

Prudent distribution

2. Conditions for distribution, access to and the use of Material and Data by researchers, including researchers employed by or affiliated with for-profit entities, should be openly stated by the Brain Bank.

Obligation to state conditions for distribution and access

3. Transfers of any Material and Data should only take place under conditions of material transfer agreement or a comparable document, which sufficiently describes the rights and obligations of the recipient party.

Obligation to conduct agreements with the recipient

Article 21 Requests for Material

Formal requirement to requests for Material and Data

1. Request for Material and Data should contain at the least the following information:

i. description of the research project;
ii. the quantity and nature of the Material and Data requested;
iii. proposed use for the Material and Data;
iv. responsible person or legal entity receiving the Material;
v. whether all required approvals, including reviews by an ethics committee or equivalent or research counsel, have been obtained;
vi. whether research has been peer-reviewed.

Proper documentation of requests

2. All received requests for Biological Material and Data should be documented and attended to.

Requests evaluation

3. All requests for Material and Data should be internally reviewed, preferably by the evaluation panel consisting of designated individuals, knowledgeable in the requestor's field of research. Any affiliations which may create a conflict of interest for the panel member should be disclosed. Any panel member with conflicts of interests regarding certain evaluation should withdraw from the panel and another member should be appointed.

Evaluation of request content

4. It should be explicitly evaluated whether the proposed use for the Material and Data falls within the scope of Informed Consent or Authorization, including all restrictions placed by the Donor, next of kin or a representative.

5. When evaluating the contents of the request the panel should consider:

i. whether the requested Material and Data are suitable for the proposed uses;
ii. whether the requested amount of Material and Data are not excessive in relation to the purposes of research;
iii. whether research is not repeated by other requestors, unless for the purpose of verification of novel findings;
iv. whether the quality of proposed research complies with scientific standards .

Scares samples

6. Distribution of scarce samples of Material and Data should be prioritized fairly and equitably.

Obligation to motivate refusal

7. If and when request for Material and Data is denied, the reasons why the request has been denied should be clearly explained to the requestor.

Article 22 Research Results

Reporting of research results

1. Brain Banks should prompt the users to report results and additional findings obtained through research on Material and Data received from the Brain Bank, as soon as possible, unless explicitly agreed otherwise with the recipient of the Material.

Communicating research results

2. Every Brain Bank should have a written policy with regard to communication of research results to the general public as well as to individuals of a certain group. In particular the future Donors of the Biological Material and their family members should be informed of such policy

Article 23 Information on Hereditary Diseases

Both post-mortem examination and research performed with the Material and Data can give rise to Information on Hereditary Diseases, whilst Informed Consent or Authorization may not contain specific instructions on how to handle such Information or may not originate from the person who is possibly affected by that Information. The partners of the BNE Consortium recognise such limitations of Informed Consent and Authorization and adhere to the guidelines stated in this article.

Genetic information

1. When the relative of the donor has explicitly requested or has given a fully informed and explicit consent to receive Information on Hereditary Diseases by which he or she is possibly affected, the Information should be communicated to that person.

Consent or request to receive information

2. When Informed Consent or Authorization is subject to limitations mentioned in paragraph 1 and Information on Hereditary Diseases emerges from post-mortem diagnostic procedures, the wish either to know or not to know that Information of the person who is possibly affected by it, should be respected. The medical doctor communicating the results of post-mortem examination, to a possibly affected relative of the donor should warn that person, that the pathology report may contain Information on Hereditary Diseases and ask whether this individual wants to hear the genetic details of the diagnostic findings.

Genetic information from autopsy findings

The right not to know and obligation to ascertain the wish to know

3. The persons possibly affected by the Information on Hereditary Diseases as stated in paragraph 2 and 3 of this article, should be referred to a specialised medical genetics centre for further diagnostics and counseling, whenever this is deemed necessary.

Referral to genetic specialists

4. When Informed Consent or Authorization is subject to limitations mentioned in paragraph 1, and Information on Hereditary Diseases emerges in the course of research performed with the Material and Data, it should be evaluated whether such Information must be communicated to the surviving relatives of the donor. Evaluation should take place by the appointed individuals of the Brain Bank in consultation with the ethics committee. In particular it should be considered:

Genetic information from research findings

i. whether the persons who are possibly affected by the Information are identifiable and reachable;
ii. whether withholding the Information on Hereditary Diseases is likely to cause damage to these individuals;
iii. whether the benefits of communicating such Information outweigh any disturbance or harm it may cause;
iv. whether there are other weighty reasons to feed-back the Information.

Once decided to communicate such Information to the persons concerned, this should be done via a family physician or a medical genetics centre, which can provide appropriate counseling and due care.

Code of Conduct
BNE Consortium
May 30th, 2008
